# Psychological intervention priorities according to perinatal women who experienced suicidal thoughts and perinatal mental health professionals: a Q-methodology study

**DOI:** 10.3389/fpsyt.2023.1286500

**Published:** 2023-12-21

**Authors:** Holly E. Reid, Daniel Pratt, Dawn Edge, Anja Wittkowski

**Affiliations:** ^1^Division of Psychology and Mental Health, School of Health Sciences, Faculty of Biology, Medicine and Health, University of Manchester, Manchester, United Kingdom; ^2^Manchester Academic Health Sciences Centre, Manchester, United Kingdom; ^3^Greater Manchester Mental Health NHS Foundation Trust, Manchester, United Kingdom

**Keywords:** mothers, staff, pregnancy, postpartum, suicide, Q-sort, support, treatment

## Abstract

**Introduction:**

Suicide is the leading direct cause of maternal death in the year following birth and the second leading cause during pregnancy, in the UK and Ireland. Currently no evidence-based psychological interventions exist specifically designed to reduce mothers’ suicidal experiences during the perinatal period. Reducing suicidal ideation and behaviour in mothers is a priority to prevent deaths and lessen the distress felt by mothers and their families. As Q-methodology measures the consensus and disagreement between individuals on a given topic, the current study used Q-methodology to elicit the priorities for a future psychological intervention aimed at reducing suicidal ideation and behaviour during the perinatal period, from the collective perspectives of both mothers and professionals.

**Method:**

As part of this Q-methodology study, we developed a Q-set of 75 statements pertaining to possible elements of a psychological intervention that might help reduce a mother’s suicidal ideation and behaviour during the perinatal period. Mothers and professionals were recruited via perinatal mental health services and social media advertisements.

**Results:**

Twenty-one mothers and 11 perinatal mental health professionals ranked each Q-set statement depending on its perceived importance in developing a new intervention. A centroid factor analysis was conducted and two factors, which accounted for 42% of the overall variance, were identified: Factor 1 *“supporting the mother to create distance between herself and the appeal of suicide”* and Factor 2 “*establishing positive connections with the therapist, the baby and motherhood*.” All participants believed that developing plans to keep the mother safe from suicide was the most important aspect for inclusion in a future intervention. Participants who loaded onto Factor 1 also prioritised supporting mothers to learn more about triggers for their suicidal ideation and behaviour. Ensuring a robust therapeutic alliance was more important for those who loaded onto Factor 2.

**Conclusion:**

This is the first study using Q-methodology to explore the psychological intervention priorities of mothers and professionals. Findings indicate clear priorities in terms of planning and coping during a crisis, endorsed by all participants, and provide an initial step in the development of a new perinatal suicide prevention intervention.

## Introduction

The most recent UK and Ireland Confidential Enquiries into Maternal Deaths and Morbidity (MBRRACE-UK) report highlighted that, during 2019 through to 2021, maternal suicide was the leading direct cause of death between 6 weeks and a year after the end of pregnancy, and the third largest direct cause of death during or within 6 weeks of the end of pregnancy (1). Since publication of the first MBRRACE-UK report outlining the 2012–2014 data, maternal suicide has been the leading direct cause of death between 6 weeks and a year after the end of pregnancy every year (1–8). Furthermore, the rates of suicide per 100,000 maternities have seen very marginal changes year-by-year over the past decade, until 2020, which saw a sharp uptick in the rates; this increase emphasises the urgent need to improve efforts to reduce maternal suicide during the perinatal period (i.e., pregnancy and the first postpartum year). Not only is it important to prevent deaths by suicide in mothers, but it is also equally vital that occurrences of suicidal ideation and behaviour are reduced due to the heightened distress these experiences cause to mothers and their families.

Currently, the National Institute for Health and Care Excellence (9) guidelines advise a high intensity psychological therapy, such as cognitive behavioural therapy (CBT) and/or antidepressant medication, for the treatment of moderate to severe depression during the perinatal period. However, there are no evidence-based guidelines for the specific targeting of suicidal ideation and behaviour in women during pregnancy or the first postpartum year. Indeed, suicidal ideation and behaviour can occur in the context of depression, but these suicidal thoughts and actions should not be treated as symptoms of depression or any other mental health condition. Thus, it is important that guidelines advising on how to treat suicidal ideation and behaviour, regardless of a psychiatric diagnosis, are developed. Although NICE (10) advises a structured, person-centred CBT-informed psychological intervention for adults who self-harm to prevent the recurrence of self-harm, these guidelines do not indicate how to reduce suicidal ideation and suicidal behaviour nor have they been developed for perinatal women. Meta-analytic systematic reviews have demonstrated that psychological and psychosocial interventions can be effective at reducing suicidal thoughts and behaviours in people with mental health problems [e.g., (11, 12)]. However, the development of psychological therapies focused on suicidal thoughts and acts based on empirically supported psychological models of suicidal thoughts and behaviours in the general population is in its infancy.

The perinatal period encompasses challenges not experienced at any other time of life, such as learning how to breastfeed (13), bonding with baby (14) and changes to the romantic relationship (15). Qualitative research with mothers who have experienced suicidal ideation during the perinatal period has also identified a wide range of factors believed to trigger and/or maintain suicidal thoughts and behaviour. For example, feeling isolated (16), feeling a loss of control (16, 17), self-appraisals of being a “bad mother” (16, 18) and the incongruence between a mother’s expectations and the reality of motherhood (16, 17) have all been included in models of suicidal ideation during the perinatal period. Due to the unique characteristics of the perinatal period and the range of perceived contributors to suicidal outcomes, it is important to determine the intervention priorities of those the intervention aims to serve and those who provide professional perinatal mental health care to suicidal mothers during the perinatal period. It is important a new intervention is not only effective at reducing suicidal ideation and behaviour, but also acceptable, in that mothers are willing to engage in the intervention and professionals are willing to be trained in and to deliver it.

There is huge variation in personal circumstances and challenges arising during the perinatal period that could contribute to a mother’s suicidal ideation and because of this the intervention priorities of suicidal mothers are potentially diverse and intricate. Q-methodology offers a useful approach to reduce the myriad perspectives on a potential intervention to a manageable number of points of view that can then be used by researchers and clinicians. Q-methodology is unique in that it requires participants to engage explicitly with opinions of which they might never have thought or might deem inappropriate or surprising, which can mitigate response bias and provide a thorough investigation of the many possible and potential priorities for a psychological intervention (19).

Q-methodology has been used to explore adolescents’ attitudes towards suicide (20) and the reasons for repeated self-harm (21). For example, Bryant et al. (21) propose that their findings can be used to individualise therapy to better meet the needs and goals of individuals who repeatedly self-harm. Furthermore, within the sphere of perinatal mental health research, Butler et al. (22) used Q-methodology to explore the acceptability and feasibility of a parenting intervention for mothers with perinatal mental health difficulties being delivered on a psychiatric inpatient Mother and Baby Unit (MBU), with samples of 15 mothers and 16 MBU staff (23).

To ensure the present study captured the priorities of those who would be the target population of the future intervention and those who could be involved in its delivery, both mothers who have experienced suicidal ideation and perinatal mental health professionals were included. Thus, using Q-methodology, we aimed to elicit the priorities for a psychological intervention aimed at reducing suicidal ideation and behaviour during the perinatal period, from the perspectives of both mothers and professionals.

## Methods

### Design

This study used Q-methodology with a sample of mothers who experienced suicidal thoughts and might have attempted suicide during the perinatal period, and also a sample of mental health professionals who have worked with suicidal mothers during the perinatal period. Q-methodology combines elements of qualitative and quantitative methods to measure subjectivity (i.e., opinions, values and beliefs) (24, 25). Unlike interviews, Q-methodology can measure the consensus and disagreement between individuals on a given topic (26). A Q-methodology study involves inverting the sample and variables, so the variables are no longer the tests or measures, rather the individuals who participate in the study become the variables of interest (19). In brief, a Q-methodology requires the development of a set of statements (termed a Q-set) that represents the diversity of opinions present on a particular topic. A group of participants (called a P-set) systematically rank these statements (each participant’s resulting sort is termed a Q-sort) (27). The correlations between participants, and not correlations between measures, are explored and therefore the analysis correlates the Q-sorts from across the P-Set and provides an indication of the similar segments of subjectivity that exist within the group of participants (25).

### Ethical considerations and research governance

The study was reviewed by a National Health Service (NHS) Research Ethics Committee (21/PR/0374) and received ethical approval from the Health Research Authority. After providing informed consent, each mother gave contact details for a healthcare professional (e.g., their general practitioner or care coordinator) that could be contacted if the participant required a health professional’s support. All participants were given contact details for mental health support before and after participation and were asked if they would like to receive a summary of the study findings upon completion of the analysis and study write-up.

### Participant eligibility criteria

#### Mothers

Mothers were included if they were aged 18 years or older and had experienced suicidal thoughts during pregnancy and/or the first postpartum 12 months. There were no limits on when a mother’s perinatal suicidal experiences occurred (i.e., mothers could have been suicidal at the time of the study or at any time in the past) in order to sample mothers who ranged in terms of recency of their suicidal thoughts. Eligible participants were also required to speak and comprehend English well enough to provide consent and complete the Q-sort, and have access to either a desktop computer, laptop, tablet or smartphone and internet connection to take part in the study procedure. If a mother could not provide contact details for a health professional that could be contacted if the participant needed further support, she was excluded from the study.

#### Professionals

Eligible mental health professionals had to have had direct contact with and provided care to women who felt suicidal during the perinatal period within relevant inpatient or community services. To be eligible, professionals were also required to have been in their post for at least 3 months to ensure they had sufficient professional experience to complete the Q-sort. Non-permanent staff, such as agency and bank staff, were excluded due to their varying levels of professional experience within perinatal services.

### Sample size considerations

Although Q-methodology embraces smaller numbers of participants, there are no stipulated thresholds for the number of participants required to perform a successful Q-methodology study. Therefore, the number of participants in the current P-set was guided by the two aforementioned similar Q-methodology studies, whereby a sample of 15 psychiatric MBU service users (22) and a sample of 16 MBU health professionals (23) were recruited to investigate the perceived acceptability of a parenting programme if delivered in an MBU setting. The P-set size is also guided by Watts and Stenner (25) who advocate that a Q-methodology should consist of fewer participants than items in the Q-set.

### Recruitment

Recruitment took place between September 2021 and January 2022.

#### Mothers

Participants were either referred to the study by staff working within an MBU in the Northwest of England, by staff working for perinatal community mental health teams in the Northwest, or self-referred via social media advertisements. Mothers who had participated in our previous study, a qualitative investigation of suicidal ideation during the perinatal period [see (16)], were also invited to participate in this current study. Participants who wished to take part were given a participant information sheet. The first author ensured potential participants had capacity to consent to take part by asking the mothers questions to assess whether they understood the information relevant to participating, could retain the information, could weigh up the risks and benefits of participating and could communicate their decision to participate. The first author also ensured that potential participants had had any questions answered before asking them to complete a consent form.

#### Professionals

The study was advertised via email communications circulated to professionals working within an MBU and perinatal community mental health teams in the Northwest of England, and via advertisements posted on social media. We also invited professionals who had participated in our previous study, a qualitative investigation of professionals’ experiences of working with mothers who are suicidal [see (28)], to take part in this current study. Professionals who wished to participate accessed the study via a link provided on the study advertisements. The link presented the participant information sheet, and participants who wished to proceed were asked to confirm that they met the eligibility criteria. To reduce the burden to professionals, staff participants were not required to complete a consent form, rather their completion of the questionnaires and Q-sort was taken as their consent to take part, as approved by the Research Ethics Committee.

### Procedure

The design and conduct of the study were primarily guided by Watts and Stenner’s (25) recommendations. We also followed the checklist developed by Dieteren et al. (29) to assist with the reporting of this Q-methodology study.

#### Concourse and Q-set development

Initially, a concourse was developed, which is a collection of statements thought to represent all viewpoints on the topic of interest (25). For the current study, the concourse comprised of statements pertaining to elements of psychological intervention and support for mothers during the perinatal period aimed at reducing suicidal ideation and behaviour. Statements were gathered by the first author from social media posts, blog posts, newspaper articles and academic literature which was searched using terms such as “suicid*” AND “mothers” OR “postpartum” OR “pregnant” OR “perinatal” combined with “intervention” OR “treatment” OR “therapy” OR “support” OR “help.” Interview transcripts from our two previous qualitative studies were also used as source material for the concourse: one of these studies involved interviewing mothers who were suicidal during the perinatal period (16) and the other involved interviewing mental health professionals about working with suicidal mothers (28).

The initial concourse comprised 112 written statements. A member of the study’s service user reference group (i.e., a mother who had experienced postpartum suicidal thoughts) volunteered to assist the first author in refining the concourse which then led to the development of a final Q-set. Firstly, the 112 items were organised into themes (there were 14 themes in total), statements that were very similar in content were either combined or removed, and each statement was then carefully considered for relevance and appropriateness to meet the study aims. The quantity of statements was reduced whilst ensuring each theme was adequately and succinctly reflected in the Q-set by at least one statement. Once the Q-set was agreed, all authors scrutinised and refined the phrasing of items, to arrive at a final Q-set of 75 written statements (see Table 1 for the final Q-set and corresponding themes). Ordinarily, the inclusion of statements that are deemed to reflect controversial viewpoints in a Q-set is encouraged. However, as we aimed to elicit priorities for an intervention, our final Q-set consisted as much of possibilities as opinions.

**Table 1:** The Q-set statements and corresponding themes.

*A psychological intervention for perinatal suicide should…*		
No.	Statement	Theme
S1	Put positive plans in place for the future	Looking to the future
S2	Deal with imagined frightening future scenarios (e.g., falling down the stairs with baby)	
S3	Involve listening to others feeling the same way	Normalising
S4	Involve listening to other women who are struggling with not sleeping/nausea/baby teething/baby illnesses	
S5	Normalise not feeling a rush of love once baby is born (e.g., it’s normal to be too exhausted to feel anything; it’s normal to feel relieved it’s over)	
S6	Involve listening to others who have felt suicidal when perinatal and got better	
S7	Encourage women to read baby books	Bonding
S8	Highlight the ways women are caring for their baby	
S9	Suggest ways of bonding that are not breastfeeding	
S10	Explore why women may feel nothing towards their baby	
S11	Encourage women to draw a map of their support network (people they can talk to or can help in some way)	Identifying support
S12	Be delivered by a therapist who has also experienced perinatal mental health difficulties	The therapist
S13	Involve someone to talk to as soon as the suicidal thoughts come back	
S14	Be delivered by the same therapist at every contact	
S15	Be delivered by a therapist who demonstrates that they understand	
S16	Involve a therapist keeping detailed notes	
S17	Include education about the psychological transition to parenthood	Psychoeducation
S18	Include education about mental health difficulties	
S19	Help women realise that having suicidal thoughts is problematic	
S20	Identify the irrational thoughts that lead to suicidal thoughts	Causes of suicidal thoughts
S21	Explore a woman’s relationships with her own parents	
S22	Challenge the idea that the baby would be better off without their mother	
S23	Challenge the thoughts of being a ‘bad mum’	
S24	Help a woman identify suicidal risk factors (e.g., having an abusive partner)	
S25	Remove shame	
S26	Determine what the suicidal thoughts are AND feelings about them	
S27	Ease anxiety about the prospect of birth or the birth experience	
S28	Be delivered in the woman’s own home	Delivery of intervention
S29	Be delivered remotely (e.g., via email, text or online chat)	
S30	Involve targeted peer support (i.e., being paired with a similar woman who no longer has suicidal thoughts)	
S31	Involve a day session where women and babies can go for a whole day and leave in the evening	
S32	Feel informal	
S33	Involve feeling looked after (e.g., lots of cups of tea and biscuits provided)	
S34	Be delivered while walking outside	
S35	Be delivered via a phone app	
S36	Focus on changing how women feel rather than just talking about how women feel	
S37	Ensure childcare is available while women access the intervention (i.e., for the baby and older siblings)	
S38	Be structured (rather than unstructured)	
S39	Be delivered as a group session (rather than one-to-one)	
S40	Highlight women’s purpose (e.g., how she is growing a baby or how she is looking after baby)	Increase a mother’s worth
S41	Explore how a woman’s purpose for being here has changed from pre-baby to present	
S42	Challenge women’s anxiety of ‘ruining the baby’s life’	
S43	Address women’s feelings of failure	
S44	Help women identify that they have a place in the world	
S45	Build confidence in baby-related tasks (e.g., changing a nappy, bathing baby)	
S46	Challenge the pursuit for perfectionism (e.g., you are good enough)	
S47	Help women feel significant again	
S48	Identify why a woman is the best mother for their baby	
S49	Help women deal with the identity change from pre-baby self to mother	Transition to motherhood
S50	Explore expectations for pregnancy/motherhood?	
S51	Help women cope with the chaotic-ness of a baby	
S52	Help women cope with not meeting their breastfeeding goals	
S53	Help women to meet their breastfeeding goals	
S54	Explore thoughts of wanting the pre-baby life back	
S55	Address that the prospect of being a mother can be frightening	
S56	Help women cope if they have feelings of not wanting to look after the baby	Tools to cope
S57	Include mindfulness	
S58	Help women to cope when feeling trapped	
S59	Put a plan in place for when the darkness descends/feeling absolutely nothing	
S60	Help women to cope with the worry of feeling suicidal	
S61	Explore catastrophising thoughts	
S62	Help women cope with the urge to harm themselves^a^	
S63	Help women manage anger	
S64	Help women to relinquish control	
S65	Explore ways to ask for attention that do not involve self-harm/attempting suicide	
S66	Put a plan in place for how to respond to suicidal thoughts when alone/with the baby only	
S67	Help women to structure their day	Tools to negotiate daily life
S68	Encourage babywearing	
S69	Help women to cope with life hurdles (e.g., child accidentally bumping their head)	
S70	Help women feel more free	Reframing suicide
S71	Challenge thoughts of suicide as a positive option	
S72	Challenge the perceived helpfulness of non-suicidal self-harming (if applicable)	
S73	Provide ways a woman can comfort herself without thinking about suicide	
S74	Ensure women have financial stability	Practical support
S75	Involve self-care activities that can be finished (e.g., baking a cake)	

a This study is focused on an intervention to reduce suicidal ideation and behaviour, rather than to reduce “self-harm” (a term which can encompass both suicidal and non-suicidal self-injury). However, when gathering statements for the Q-set, it became apparent that when in crisis some mothers described an urgent need to harm themselves in such a way that they would die, rather than describing an explicit want to attempt suicide (i.e., harming herself in a fatal way was more important than dying at that moment). Therefore, we have included the statement “help women cope with the urge to harm themselves” (S62) to better reflect the urge that mothers might feel when in crisis.

#### Q-sorting

The study was administered online using the Qualtrics™ survey platform, and participants were given the choice as to whether they completed the study with the assistance of the first author in attendance via telephone or videoconferencing software (e.g., Zoom^™^), or on their own. Two pilot Q-sorts were completed by two mothers, one of which had experienced suicidal thoughts during pregnancy. These pilots a) identified that the wording of some statements required amending, b) identified technical problems, c) confirmed the suitability of the instructions to complete the Q-sort without researcher assistance, and d) confirmed the inclusivity of different views, preferences and needs within the Q-set.

**Figure 1:**
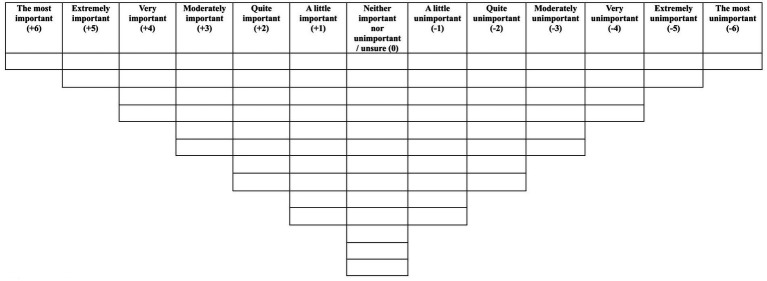
Forced choice response grid.

Prior to completing the Q-sort, participants provided demographic information. For mothers this included their age, ethnicity, how many children they had and information regarding their suicidal thoughts and behaviours, and for staff this included their ethnicity and details about their professional post and experience. Each participant was presented with the 75 statements in a random order and asked to rank the statements based on how important they believed that particular statement to be in a psychological intervention to reduce suicidal thoughts and behaviour during the perinatal period. Initially, participants sorted the statements into three categories: important, neutral and unimportant. Participants were then asked to use these three categories to assist them in systematically ranking the statements from “the most important” to “the most unimportant,” by sorting them into a forced choice distribution grid (see Figure 1). Participants were given the choice to review their Q-sort configuration and make amendments if they believed that they had made a mistake. Each participant was also asked to complete a post-sort questionnaire, which was administered to a) gain insight into why participants ranked the particular statements at the extreme ends of the grid, b) understand better whether there were any statements participants did not understand and c) see whether participants believed that any other aspects of psychological support for women feeling suicidal during the perinatal period were absent from the Q-set.

### Data analysis

The analysis was conducted using PQMethod version 2.35 (30), a statistical package developed specifically for analysing Q-methodology data. Once each Q-sort had been entered into PQMethod, the data were subjected to a centroid factor analysis (31, 32). Principal component analysis offers another data extraction method whereby the mathematically best solution will be presented, whereas centroid factor analysis leaves all possible solutions open and therefore provides an opportunity for the researcher(s) to properly engage with the data and explore the possible solutions through factor rotation. It is for these reasons that Watts and Stenner (25) recommend centroid factor analysis as the preferable data extraction method and why we chose it over principal component analysis for this study. Initially, PQMethod creates a correlation matrix which presents the intercorrelations between each of the Q-sort configurations. The software then extracts factors from this correlation matrix, with each factor reflecting a portion of common variance (i.e., a shared opinion). Therefore, participants that ranked the statements in a similar way would be clustered together and load onto the same factor (31). There may be multiple possibilities when instructing the software on how many factors to extract and our decision was guided by the Kaiser-Guttman criterion (i.e., only factors with an eigenvalue greater than 1 were extracted) [see (33–35)]. A varimax rotation was performed on the unrotated factors to maximise the amount of variance explained by each factor. In a varimax rotation, the software automatically conducts the rotation using statistical criteria and this was chosen over a manual ‘by-hand’ rotation, due to varimax rotation being the favoured option when conducting a more inductive analysis (25).

Significant factor loadings were calculated for the *p* < 0.01 level using the equation of 2.58(1/*√number of items in the Q-set) = 2.58*(1/*√75) = 0.30* (25). Factor arrays were then created for each of the two factors; an exemplar Q-sort which represents the viewpoint of the factor. The PQMethod software created these arrays using weighted averages of each of the Q-sorts that significantly loaded onto the factor. The software also calculates the correlation between the factor scores using the Pearson correlation coefficient. Once the centroid factor analysis was completed and the factors decided upon, the interrelationship of the statements within the factor array, demographic information of those participants’ Q-sorts that loaded significantly onto the factor and their post-sort questionnaire responses, along with the distinguishing statements, were used to provide a holistic interpretation of the factor; a “new gestalt” (31, 36). The interpretation was assisted through the development of a crib sheet [outlined in (25)] which forced the first author to engage with and compare the position of each statement in the factor arrays. This engagement was driven by the logic of abduction (31, 37, 38), whereby the first author continually questioned what the position of each statement was trying to tell her. In practise, this involved the first author questioning the ranking of each statement in the factor array and then creating a preliminary hypothesis. This process was repeated for each statement in the factor array along with the first author frequently “zooming out” to think about the whole viewpoint reflected by the factor array (25).

## Results

### Participant characteristics

Forty-two mothers initially expressed an interest in participating, of whom 16 became uncontactable and five later withdrew from participation. Reasons for withdrawal included feeling uncomfortable providing contact details of a named healthcare professional and fear of reliving traumatic memories. Of the final 21 mothers who completed the Q-sort, 18 found out about the study via a social media advertisement, two were participants of a previous study run by the research team and notified of this current study via email, and one heard about the study through word of mouth.

A total of 15 professionals started the Q-sort, four did not complete it, leaving a total of 11 professionals who completed the Q-sort. Therefore, the P-set comprised a total of 32 participants who completed the Q-sort: 21 mothers and 11 professionals. The P-set’s demographic data can be found in Table 2.

**Table 2:** Participants’ demographic information.

Mothers (*n* = 21)	
Age (years)	Range: 28–64; Mean: 40.5
Ethnicity	17 White British (81%), 1 White American (5%), 1 Asian/Asian British Indian (5%), 1 Asian/Asian British Pakistani (5%), 1 Black/Black British African (5%)
Sexual orientation	15 heterosexual (71%), 5 bisexual (24%), 1 prefer not to say (5%)
Marital status	17 married/living together (81%), 3 single (14%), 1 living separately (5%)
Highest level of education	1 GCSEs/CSEs/O-levels (5%), 1 A-levels/BTEC (5%) 10 Undergraduate degree (48%), 8 Postgraduate degree (38%), 1 Doctorate degree (5%)
Main employment status	8 full-time (38%), 7 part-time (33%), 3 homemaker (14%), 1 carer (5%), 1 retired (5%), 1 unemployed (5%)
Last felt suicidal during the perinatal period	2 currently or within the last week (10%), 3 within the last 3 months but not currently (14%), 2 between 3 and < 12 months ago (10%), 2 between 1 and < 2 years ago (10%), 2 between 2 and < 5 years ago (10%), 3 between 5 and < 10 years ago (14%), 7 10 or more years ago (33%)
When suicidal thoughts were experienced	3 pregnancy only (14%), 9 postpartum only (43%), 6 pregnancy and postpartum (29%), 1 childbirth and postpartum (5%), 2 pregnancy, childbirth and postpartum (10%)
Suicidal thoughts, planning or attempted suicide	11 had thoughts alone (52%), 9 had thoughts and planned (43%), 1 had thoughts, planned and attempted (5%)
Number of children	2 currently pregnant and no other children (10%), 11 one child (52%), 5 two children (24%), 2 three children (10%), 1 four children (5%)
Age of children (years)	Range: 1–31; Mean: 11.2

Professionals (*n* = 11)	
Gender	1 male (9%), 10 female (91%)
Ethnicity	8 White British (73%), 2 Mixed White and Black Caribbean (18%), 1 White Irish (9%)
Professional role	3 perinatal mental health nurses (27%), 2 perinatal clinical psychologists (18%), 2 perinatal peer supporters (18%), 1 perinatal occupational therapist (9%), 1 psychiatry trainee (9%), 1 counsellor (9%), 1 support worker (9%)
Duration spent working with perinatal women (years)	Range: 1–24; Mean: 7.5

### Q-sort analysis and interpretation

Significant factor loadings were calculated for the *p* < 0.01 level using the equation of 2.58(1/*√number of items in the Q-set) = 2.58* (1/*√75) = 0.30* (25). However, this resulted in 21 of the Q-sorts loading significantly onto more than one factor (i.e., they were confounded Q-sorts). In order to arrive at a solution whereby more of the Q-sorts loaded onto just one of the factors significantly (i.e., fewer confounded Q-sorts), the significant factor loading was increased from 0.30 to 0.45, in line with Watts and Stenner’s (25) recommendation. This resulted in a more useful solution, with 25 Q-sorts loading significantly onto one of the factors, only two confounded Q-sorts and five Q-sorts that did not load significantly onto any factor (i.e., non-loading Q-sorts).

**Figure 2:**
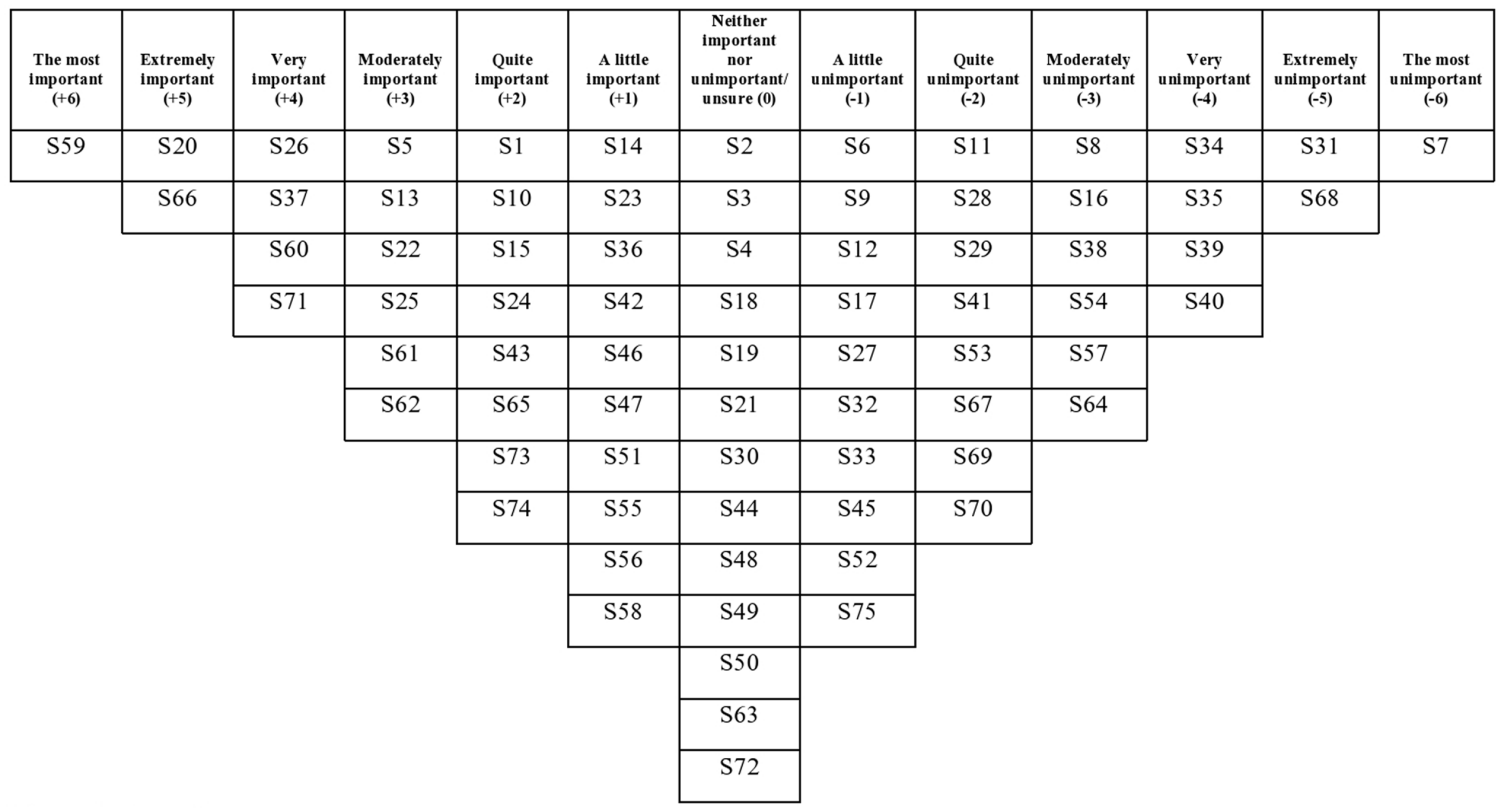
Factor 1 array.

**Figure 3:**
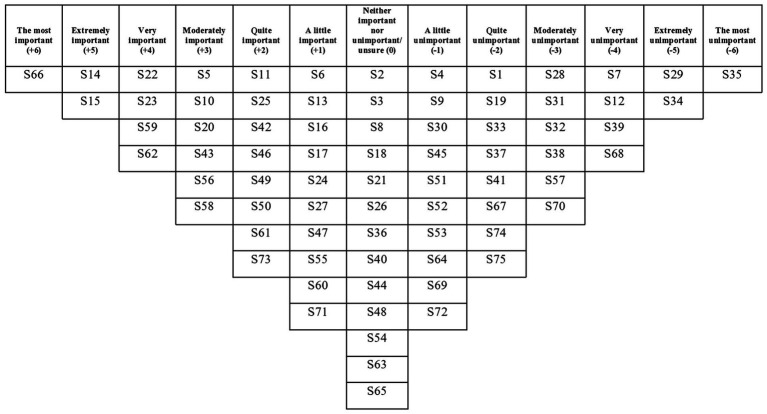
Factor 2 array.

The two factors had eigenvalues over 1, which satisfied the Kaiser-Guttman criterion, so these were the only two factors that were extracted and rotated. The two factors explained a total of 42% of the study variance. There were no bipolar factors (i.e., a factor that has both positively and negatively loading Q-sorts and therefore contains two opposing viewpoints). There was a strong correlation between Factors 1 and 2 (*r* = 0.71), which suggests overlap between the viewpoints captured by each factor. See Table 3 for each written Q-set statement along with the corresponding factor array rankings, and Figures 2, 3 for the Factor 1 and 2 factor arrays, respectively, displayed in the response grids. The interpretations of each factor and demographic details of the participants whose Q-sorts significantly loaded onto each factor are described below, and pertinent quotations from participants’ post-sort questionnaire responses are provided to support the interpretation.

**Table 3:** Factor arrays for Factors 1 and 2.

No.	Statement	Factor arrays	
		F1	F2
S1	Put positive plans in place for the future	+2	-2
S2	Deal with imagined frightening future scenarios (e.g., falling down the stairs with baby)	0	0
S3	Involve listening to others feeling the same way	0	0
S4	Involve listening to other women who are struggling with not sleeping/nausea/baby teething/baby illnesses	0	-1
S5	Normalise not feeling a rush of love once baby is born (e.g., it’s normal to be too exhausted to feel anything; it’s normal to feel relieved it’s over)	+3	+3
S6	Involve listening to others who have felt suicidal when perinatal and got better	-1	+1
**S7**	**Encourage women to read baby books**	**−6**	−4
S8	Highlight the ways women are caring for their baby	−3	0
S9	Suggest ways of bonding that are not breastfeeding	−1	−1
S10	Explore why women may feel nothing towards their baby	+2	+3
S11	Encourage women to draw a map of their support network (people they can talk to or can help in some way)	−2	+2
S12	Be delivered by a therapist who has also experienced perinatal mental health difficulties	−1	−4
S13	Involve someone to talk to as soon as the suicidal thoughts come back	+3	+1
S14	Be delivered by the same therapist at every contact	+1	+5
S15	Be delivered by a therapist who demonstrates that they understand	+2	+5
S16	Involve a therapist keeping detailed notes	−3	+1
S17	Include education about the psychological transition to parenthood	−1	+1
S18	Include education about mental health difficulties	0	0
S19	Help women realise that having suicidal thoughts is problematic	0	−2
S20	Identify the irrational thoughts that lead to suicidal thoughts	+5	+3
S21	Explore a woman’s relationships with her own parents	0	0
S22	Challenge the idea that the baby would be better off without their mother	+3	+4
S23	Challenge the thoughts of being a ‘bad mum’	+1	+4
S24	Help a woman identify suicidal risk factors (e.g., having an abusive partner)	+2	+1
S25	Remove shame	+3	+2
S26	Determine what the suicidal thoughts are AND feelings about them	+4	0
S27	Ease anxiety about the prospect of birth or the birth experience	−1	+1
S28	Be delivered in the woman’s own home	−2	−3
S29	Be delivered remotely (e.g., via email, text or online chat)	−2	−5
S30	Involve targeted peer support (i.e., being paired with a similar woman who no longer has suicidal thoughts)	0	−1
S31	Involve a day session where women and babies can go for a whole day and leave in the evening	−5	−3
S32	Feel informal	−1	−3
S33	Involve feeling looked after (e.g., lots of cups of tea and biscuits provided)	−1	−2
S34	Be delivered while walking outside	−4	−5
**S35**	**Be delivered via a phone app**	−4	**−6**
S36	Focus on changing how women feel rather than just talking about how women feel	+1	0
S37	Ensure childcare is available while women access the intervention (i.e., for the baby and older siblings)	+4	−2
S38	Be structured (rather than unstructured)	−3	−3
S39	Be delivered as a group session (rather than one-to-one)	−4	−4
S40	Highlight women’s purpose (e.g., how she is growing a baby or how she is looking after baby)	−4	0
S41	Explore how a woman’s purpose for being here has changed from pre-baby to present	−2	−2
S42	Challenge women’s anxiety of ‘ruining the baby’s life’	+1	+2
S43	Address women’s feelings of failure	+2	+3
S44	Help women identify that they have a place in the world	0	0
S45	Build confidence in baby-related tasks (e.g., changing a nappy, bathing baby)	−1	−1
S46	Challenge the pursuit for perfectionism (e.g., you are good enough)	+1	+2
S47	Help women feel significant again	+1	+1
S48	Identify why a woman is the best mother for their baby	0	0
S49	Help women deal with the identity change from pre-baby self to mother	0	+2
S50	Explore expectations for pregnancy/motherhood?	0	+2
S51	Help women cope with the chaotic-ness of a baby	+1	−1
S52	Help women cope with not meeting their breastfeeding goals	−1	−1
S53	Help women to meet their breastfeeding goals	−2	−1
S54	Explore thoughts of wanting the pre-baby life back	−3	0
S55	Address that the prospect of being a mother can be frightening	+1	+1
S56	Help women cope if they have feelings of not wanting to look after the baby	+1	+3
S57	Include mindfulness	−3	−3
S58	Help women to cope when feeling trapped	+1	+3
**S59**	**Put a plan in place for when the darkness descends/feeling absolutely nothing**	**+6**	+4
S60	Help women to cope with the worry of feeling suicidal	+4	+1
S61	Explore catastrophising thoughts	+3	+2
S62	Help women cope with the urge to harm themselves	+3	+4
S63	Help women manage anger	0	0
S64	Help women to relinquish control	−3	−1
S65	Explore ways to ask for attention that do not involve self-harm/attempting suicide	+2	0
**S66**	**Put a plan in place for how to respond to suicidal thoughts when alone/with the baby only**	+5	**+6**
S67	Help women to structure their day	−2	−2
S68	Encourage babywearing	−5	−4
S69	Help women to cope with life hurdles (e.g., child accidentally bumping their head)	−2	−1
S70	Help women feel more free	−2	−3
S71	Challenge thoughts of suicide as a positive option	+4	+1
S72	Challenge the perceived helpfulness of non-suicidal self-harming (if applicable)	0	−1
S73	Provide ways a woman can comfort herself without thinking about suicide	+2	+2
S74	Ensure women have financial stability	+2	−2
S75	Involve self-care activities that can be finished (e.g., baking a cake)	−1	−2

Bold type highlights extremely ranked statements.

### Factor 1: Supporting the mother to create distance between herself and the appeal of suicide

#### Demographics

Thirteen Q-sorts loaded onto Factor 1, which had an eigenvalue of 12.02 and accounted for 38% of the study variance. All 13 participants that loaded onto this factor were mothers (i.e., none of the professionals’ Q-sorts loaded onto this factor), with ages ranging from 34 to 64 years (mean 44 years) and ten mothers were married or cohabiting. In terms of their suicidal thoughts, participants ranged in how recently they had experienced suicidal thoughts from experiencing suicidal ideation at the time of participation to experiencing thoughts over 10 years ago. Six mothers indicated that they had experienced only suicidal thoughts, six had experienced thoughts and engaged in suicidal planning and one had previously made a suicide attempt. Twelve of the participants that loaded onto this factor experienced suicidal thoughts during the postpartum period (six during the postpartum period only and six during pregnancy and the postpartum period) and just one participant experienced suicidal thoughts during pregnancy only.

#### Interpretation

Mothers who loaded onto this factor viewed a psychological intervention to reduce suicidal ideation and behaviour as needing to support her to create distance between herself and the appeal of suicide in a practicable way. Developing a plan to keep the mother safe was deemed the most important element of an intervention, followed by helping mothers to learn more about triggers for her suicidal ideation and behaviour. The mothers did not endorse elements that could drive feelings of inadequacy.

This viewpoint prioritised keeping mothers safe through planning for crises: “put a plan in place for when the darkness descends/feeling absolutely nothing” (S59, +6), “involve someone to talk to as soon as the suicidal thoughts come back” (S13, +3), and/or when alone: “put a plan in place for how to respond to suicidal thoughts when alone/with the baby only” (S66, +5) as the most important aspects of an intervention. Over half of the mothers who loaded onto this factor had engaged in some kind of suicidal planning (including one mother who attempted suicide) which could explain why having a plan when feeling at risk was ranked so highly in this viewpoint. Having a plan to keep mothers safe was cited as a way of preventing a suicide attempt: *“this is important because it might help from someone taking the next step when they are feeling suicidal” (Mother 14, suicidal thoughts only).* This planning to keep a mother safe could also have a positive impact on a mother’s feelings: *“a plan would have helped me feel better” (Mother 15, suicidal thoughts and planning)*. Mothers who loaded onto this factor ranged in how recently they had experienced suicidal ideation, and therefore even those who had not been in a suicidal crisis for a long time (i.e., over ten years ago) viewed planning as the priority for a psychological intervention. Planning positively for the future, not just for in times of crises, was also endorsed by this factor: “put positive plans in place for the future” (S1, +2) which significantly differentiated from Factor 2 which saw this statement ranked at (−2). Positive planning for the future could impact a mother’s sense of hope and might therefore offer an indirect effect upon reducing the suicidal ideation and behaviour: *“I feel that a sense of hopelessness about the future may be a major factor in why women feel suicidal. Having realistic but positive plans for the future would stimulate optimistic thoughts and build hope and trust in life” (Mother 7, suicidal thoughts only).*

As the participants that loaded onto this factor were all mothers and most had experienced suicidal ideation during the postpartum period, it is understandable that these mothers endorsed the need for childcare: “ensure childcare is available while women access the intervention (i.e., for the baby and older siblings)” (S37, +4), unlike Factor 2 which ranked this statement (−2). Childcare provision while accessing an intervention was cited as having an impact on the effectiveness of an intervention: *“To be effective it would be helpful if there wasn’t the distraction of a baby or worry about how to access help during school hours” (Mother 6, suicidal thoughts and planning).*

Of all the statements relating to the delivery of the intervention (rather than intervention content), ensuring availability of childcare support was ranked the most important and “involve a day session where women and babies can go for a whole day and leave in the evening” (S31, −5) was ranked the most unimportant. Reasons for this ranking included childcare difficulties: *“it’s not practical, other children cannot be involved” (Mother 17, suicidal thoughts only)* and the desire to access an intervention without children present: *“I need to work on myself alone and I already juggle that around the kids physically and emotionally” (Mother 6, suicidal thoughts and planning).*

With regards to the content of the intervention, this viewpoint emphasised the importance of helping mothers to learn more about triggers for suicidal ideation and behaviour, thereby helping develop a better understanding of this distressing experience, for example: “identify the irrational thoughts that lead to suicidal thoughts” (S20, +5), and “determine what the suicidal thoughts are AND feelings about them” (S26, +4). Following this learning, it was deemed important that these negative thoughts, feelings and beliefs were challenged: “challenge the idea that the baby would be better off without their mother” (S22, +3) and “remove shame” (S25, +3). Some mothers described that this shame arose from feeling like *“an inadequate parent” (Mother 12, suicidal thoughts and planning)* and *“failing at* var*ious aspects of pregnancy and motherhood” (Mother 11, suicidal thoughts and planning)*. Perception of inadequacy was also cited as the reason for ranking “encourage women to read baby books” (S7, −6) as the most unimportant statement for this factor: *“Women who are already floundering might find themselves feeling even more inadequate because they do not feel that they measure up to an arbitrary standard in a book” (Mother 11, suicidal thoughts and planning)*.

Helping mothers to recognise their value, which might help to combat these feelings of inadequacy, was deemed unimportant: “highlight women’s purpose (e.g., how she is growing a baby or how she is looking after baby)” (S40, −4), and “highlight the ways women are caring for their baby” (S8, −3), which significantly differed from Factor 2 which saw both of these statements ranked (0).

Along with this viewpoint’s prioritisation of planning to keep the mother safe from suicide, the overall need to support the mother to create distance between herself and the appeal of suicide through offering a greater choice of coping strategies was highlighted by the successional ranking of statements relating to the direct targeting of suicidal ideation and behaviour: “challenge thoughts of suicide as a positive option” (S71, +4), “help women cope with the urge to harm themselves” (S62, +3), and “provide ways a woman can comfort herself without thinking about suicide” (S73, +2). These rankings suggest that an intervention must prioritise targeting the idea that suicide would bring a positive outcome to a mother’s situation, then focus on finding coping strategies to replace engaging in suicidal behaviour and having suicidal thoughts.

### Factor 2: Establishing positive connections with the therapist, the baby and motherhood

#### Demographics

Factor 2 had an eigenvalue of 1.37 and accounted for 4% of the study variance. Twelve Q-sorts loaded onto this factor, nine of which were completed by professionals and three by mothers. The professionals were eight females and one male, comprised of three perinatal mental health nurses, two perinatal clinical psychologists, two perinatal peer supporters, one psychiatry trainee and one support worker. The duration of time they had been working directly with mothers during the perinatal period ranged from 1 year and 1 month to 24 years and 4 months (mean 8 years and 3 months). The three mothers ranged in age from 28 years to 42 years (mean 36 years): two experienced only suicidal ideation and one also engaged in suicidal planning. In addition, two of the three mothers experienced suicidal ideation during the postpartum period, whereas the other was pregnant. The mothers ranged widely by how recently they had experienced suicidal ideation: one reported experiencing suicidal thoughts within the 3 months prior to but not at the time of study participation, one mother between five and 10 years prior and one was over 10 years prior to participating in this study.

#### Interpretation

This viewpoint endorsed statements that resulted in positive connections for mothers when accessing a psychological intervention for suicidal ideation and behaviour during the perinatal period. Participants that loaded onto this factor particularly valued the connection with the therapist, the baby, and with motherhood more generally. The viewpoint found statements that distanced mothers from in-person therapy to be unimportant.

Similarly, to Factor 1, making plans to keep the mother safe was deemed the most important element of an intervention but this viewpoint prioritised planning for when alone: “put a plan in place for how to respond to suicidal thoughts when alone/with the baby only” (S66, +6). The majority of participants that loaded onto this factor were professionals, including a psychiatry trainee and clinical psychologists, whose jobs involve helping mothers to keep safe and well for the long-term: “*It’s important women know what to do to help themselves feel safe when alone in the community. There is always the possibility women feel suicidal again in the future, so making sure they have tools to manage this when professionals are not present is essential for the long-term” (Psychiatry trainee).*

Aside from planning to keep the mother safe, according to this viewpoint therapeutic connection took precedence over the content of the intervention: “be delivered by the same therapist at every contact” (S14, +5) and “be delivered by a therapist who demonstrates that they understand” (S15, +5). Given the high proportion of professionals who loaded onto this factor, the perceived importance of the therapist is understandable. Being seen by the same therapist enabled *“a trusting and consistent relationship” (Mother 1, suicidal thoughts and planning)* and meant mothers would not have to repeat themselves which could hinder their recovery: *“Telling even one professional that you feel suicidal when pregnant is mortifying and brings up such strong feelings of shame […] having to repeat the same story to different professionals would not be therapeutic at all” (Mother 4, suicidal thoughts only).* Similarly, this viewpoint assigned some value to therapist record-keeping: “involve a therapist keeping detailed notes” (S16, +1) which could also help facilitate the therapeutic alliance. This significantly differentiated from Factor 1 which saw this statement ranked at (−3).

Moreover, lack of connection with a therapist was not endorsed by participants who loaded onto this factor: “be delivered via a phone app” (S35, −6) and “be delivered remotely (e.g., via email, text or online chat)” (S29, −5). As well as lacking the comfort and empathy that a human can provide, accessing an intervention delivered by a phone app(lication) was viewed as being *“easily forgotten or not prioritised [by mothers]” (Mother 19, suicidal thoughts only)*.

This viewpoint also endorsed statements that could result in the strengthening of a mother’s connection with the baby and with motherhood. Challenging negative thoughts about being a mother: “challenge the idea that the baby would be better off without their mother” (S22, +4) and “challenge the thoughts of being a ‘bad mum’” (S23, +4), were viewed as very important for an intervention because professionals recalled experiences of caring for mothers who cited these as reasons for wanting to die, for example: *“Lots of women I speak to who have attempted suicide said they felt like a bad mother so I think working out why that is and helping them realise that that’s not the case is really important” (Perinatal mental health nurse 1).*

With regards to strengthening the connection between mother and baby, this viewpoint also favoured exploring unexpected feelings related to motherhood as important content to be included in an intervention: “normalise not feeling a rush of love once baby is born (e.g., it’s normal to be too exhausted to feel anything; it’s normal to feel relieved it’s over)” (S5, +3), “explore why women may feel nothing towards their baby” (S10, +3) and “help women cope if they have feelings of not wanting to look after the baby” (S56, +3).

A mother’s connection with other mothers was regarded relatively neutrally by this viewpoint: “involve listening to others feeling the same way” (S3, 0), “involve listening to other women who are struggling with not sleeping/nausea/baby teething/baby illnesses” (S4, −1), “involve listening to others who have felt suicidal when perinatal and got better” (S6, +1) and “involve targeted peer support (i.e., being paired with a similar woman who no longer has suicidal thoughts)” (S30, −1). These four statements were also ranked relatively neutrally by participants that loaded onto Factor 1. However, helping mothers identify their support network was valued slightly more: “encourage women to draw a map of their support network (people they can talk to or can help in some way)” (S11, +2), which significantly differentiated from Factor 1 which saw this statement ranked at (−2). The rankings of these statements suggest that forging new connections between the mother and other mothers as part of an intervention is not a priority but assisting the mother to identify pre-existing sources of support and then allowing her to reach out to these sources on her own terms was viewed as slightly more important.

Statements relating to a mother’s context when accessing support, such as “ensure childcare is available while women access the intervention (i.e., for the baby and older siblings)” (S37, −2) and “ensure women have financial stability” (S74, −2), were regarded as significantly less important compared to those participants who loaded onto Factor 1, which saw these statements ranked at (+4) and (+2), respectively. These differential rankings support the view that Factor 2 advocates for an intervention that prioritises ensuring a mother can connect when alone or alone with the baby and in crisis, as well as focusing on a mother’s connection with her baby and motherhood as areas to challenge and explore with a familiar and trusted therapist.

### Non-loaders

Five Q-sorts did not load significantly onto either of the factors, that is these Q-sorts did not have enough in common with either of the extracted factors and therefore these Q-sorts did not contribute to the final two factors. Four of these Q-sorts were completed by mothers and one by a professional, a perinatal occupational therapist. Three of these mothers experienced suicidal thoughts during pregnancy and the postpartum period and one mother was pregnant with her first child when she participated. None of the mothers had attempted suicide.

### Consensus statements

Statements that were not ranked statistically differently (i.e., *p* > 0.05) are known as consensus statements, and hence both factors valued these statements in a very similar way. A cross-factor comparison identified 32 consensus statements, 43% of the Q-set (see Table 4 for all 32 consensus statements). Eight of these consensus statements are particularly interesting because they were given more extreme rankings (i.e., > +3 or < −3). Respondents consistently agreed that it was important that an intervention should “normalise not feeling a rush of love once baby is born (e.g., it’s normal to be too exhausted to feel anything; it’s normal to feel relieved it’s over)” (S5, +3, +3), “help women cope with the urge to harm themselves” (S62, +3, +4), and “put a plan in place for how to respond to suicidal thoughts when alone/with the baby only” (S66, +5, +6). In contrast, the following statements were ranked similarly across factors as being unimportant for inclusion in an intervention: “be delivered while walking outside” (S34, −4, −5), “be structured (rather than unstructured)” (S38, −3, −3), “be delivered as a group session (rather than one-to-one)” (S39, −4, −4), “include mindfulness” (S57, −3, −3), and “encourage babywearing” (S68, −5, −4).

**Table 4:** Consensus statements (non-significant at *p* > 0.05).

No.	Statement	Factor 1		Factor 2	
		Rank	Z-score	Rank	Z-score
S2	Deal with imagined frightening future scenarios (e.g., falling down the stairs with baby)	0	0.09	0	−0.07
S3	Involve listening to others feeling the same way	0	−0.22	0	−0.03
S4	Involve listening to other women who are struggling with not sleeping/nausea/baby teething/baby illnesses	0	0.07	0	−0.28
S5	Normalise not feeling a rush of love once baby is born (e.g., it’s normal to be too exhausted to feel anything; it’s normal to feel relieved it’s over)	+3	1.06	+3	1.13
S9	Suggest ways of bonding that are not breastfeeding	−1	−0.30	−1	−0.30
S21	Explore a woman’s relationships with her own parents	0	−0.30	0	−0.14
S30	Involve targeted peer support (i.e., being paired with a similar woman who no longer has suicidal thoughts)	0	−0.26	−1	0.30
S33	Involve feeling looked after (e.g., lots of cups of tea and biscuits provided)	−1	−0.41	−2	−0.69
S34	Be delivered while walking outside	−4	−1.86	−5	−2.19
S36	Focus on changing how women feel rather than just talking about how women feel	+1	0.18	0	0.04
S38	Be structured (rather than unstructured)	−3	−1.22	−3	−1.23
S39	Be delivered as a group session (rather than one-to-one)	−4	−1.39	−4	−1.41
S41	Explore how a woman’s purpose for being here has changed from pre-baby to present	−2	−0.56	−2	−0.50
S42	Challenge women’s anxiety of ‘ruining the baby’s life’	+1	0.27	+2	0.64
S43	Address women’s feelings of failure	+2	0.88	+3	1.17
S44	Help women identify that they have a place in the world	0	−0.02	0	−0.04
S45	Build confidence in baby-related tasks (e.g., changing a nappy, bathing baby)	−1	−0.41	−1	−0.37
S47	Help women feel significant again	+1	0.42	+1	0.37
S48	Identify why a woman is the best mother for their baby	0	0.16	0	0.07
S52	Help women cope with not meeting their breastfeeding goals	−1	−0.44	−1	−0.24
S55	Address that the prospect of being a mother can be frightening	+1	0.27	+1	0.21
S57	Include mindfulness	−3	−1.18	−3	−1.07
S61	Explore catastrophising thoughts	+3	0.93	+2	0.88
S62	Help women cope with the urge to harm themselves	+3	1.34	+4	1.43
S63	Help women manage anger	0	0.01	0	−0.13
S66	Put a plan in place for how to respond to suicidal thoughts when alone/with the baby only	+5	1.96	+6	2.17
S67	Help women to structure their day	−2	−0.73	−2	−0.77
S68	Encourage babywearing	−5	−1.97	−4	−1.67
S70	Help women feel more free	−2	0.85	−3	0.95
S72	Challenge the perceived helpfulness of non-suicidal self-harming (if applicable)	0	−0.10	−1	0.27
S73	Provide ways a woman can comfort herself without thinking about suicide	+2	0.93	+2	0.95
S75	Involve self-care activities that can be finished (e.g., baking a cake)	−1	−0.45	−2	−0.75

### Distinguishing statements

A cross-factor comparison identified 30 distinguishing statements, 40% of the Q-set; these are statements that are ranked significantly differently in the two factor arrays at the *p* < 0.01 level (see Table 5 for all 30 distinguishing statements). Seven of these distinguishing statements are particularly interesting in that they were ranked positively (i.e., ≥ +1, indicating importance) for one factor and negatively (i.e., ≤ −1, indicating unimportance) for the other factor, these include the following: “put positive plans in place for the future” (S1, +2, −2), “involve listening to others who have felt suicidal when perinatal and got better” (S6, −1, +1), “encourage women to draw a map of their support network (people they can talk to or can help in some way)” (S11, −2, +2), “involve a therapist keeping detailed notes” (S16, −3, +1), “ease anxiety about the prospect of birth or the birth experience” (S27, −1, +1), “ensure childcare is available while women access the intervention (i.e., for the baby and older siblings)” (S37, +4, −2), and “ensure women have financial stability” (S74, +2, −2).

**Table 5:** Distinguishing statements (significant at *p* < 0.01).

No.	Statement	Factor 1		Factor 2	
		Rank	Z-score	Rank	Z-score
S1	Put positive plans in place for the future	+2	0.74	−2	−0.46
S6	Involve listening to others who have felt suicidal when perinatal and got better	−1	−0.35	+1	0.39
S7	Encourage women to read baby books	−6	−2.59	−4	−1.98
S8	Highlight the ways women are caring for their baby	−3	−1.10	0	0.11
S11	Encourage women to draw a map of their support network (people they can talk to or can help in some way)	−2	−0.51	+2	0.67
S12	Be delivered by a therapist who has also experienced perinatal mental health difficulties	−1	−0.50	−4	−1.72
S13	Involve someone to talk to as soon as the suicidal thoughts come back	+3	1.50	+1	0.35
S14	Be delivered by the same therapist at every contact	+1	0.44	+5	2.05
S15	Be delivered by a therapist who demonstrates that they understand	+2	0.67	+5	1.60
S16	Involve a therapist keeping detailed notes	−3	−1.00	+1	0.60
S20	Identify the irrational thoughts that lead to suicidal thoughts	+5	1.82	+3	1.22
S23	Challenge the thoughts of being a ‘bad mum’	+1	0.57	+4	1.43
S26	Determine what the suicidal thoughts are AND feelings about them	+4	1.52	0	0.13
S27	Ease anxiety about the prospect of birth or the birth experience	−1	−0.39	+1	0.46
S28	Be delivered in the woman’s own home	−2	−0.50	−3	−1.33
S29	Be delivered remotely (e.g., via email, text or online chat)	−2	−0.62	−5	−2.30
S31	Involve a day session where women and babies can go for a whole day and leave in the evening	−5	−1.93	−3	−1.37
S35	Be delivered via a phone app	−4	−1.44	−6	−2.66
S37	Ensure childcare is available while women access the intervention (i.e., for the baby and older siblings)	+4	1.57	−2	−0.49
S40	Highlight women’s purpose (e.g., how she is growing a baby or how she is looking after baby)	−4	−1.25	0	0.11
S46	Challenge the pursuit for perfectionism (e.g., you are good enough)	+1	0.30	+2	0.86
S49	Help women deal with the identity change from pre-baby self to mother	0	−0.13	+2	0.77
S50	Explore expectations for pregnancy/motherhood?	0	−0.25	+2	0.70
S54	Explore thoughts of wanting the pre-baby life back	−3	−0.99	0	0.08
S60	Help women to cope with the worry of feeling suicidal	+4	1.53	+1	0.44
S64	Help women to relinquish control	−3	−1.17	−1	−0.33
S65	Explore ways to ask for attention that do not involve self-harm/attempting suicide	+2	0.86	0	0.00
S69	Help women to cope with life hurdles (e.g., child accidentally bumping their head)	−2	−0.88	−1	−0.27
S71	Challenge thoughts of suicide as a positive option	+4	1.74	+1	0.24
S74	Ensure women have financial stability	+2	0.86	−2	−0.38

### Q-set feedback

In response to whether participants thought that any statements were missing from the Q-set, four mothers reported that they hesitated seeking support when experiencing suicidal ideation and consequently one or more statement(s) should have described a way to encourage mothers to seek support or to facilitate the identification of mothers who need mental health support. The mothers reported that their hesitancy to seek support was driven by the fear that alerting services to their mental health difficulties would result in the removal of their child(ren) or because they were concerned about *“how much they could trust their GP or midwife” (Mother 17, suicidal thoughts only)*. We did not include any statements related to seeking support or identifying those who would benefit from support because this was beyond the aims and scope of this study, which was namely to focus on the content of an intervention and its possible delivery.

Babywearing refers to the practise of carrying a baby in a sling or baby carrier; however, three mothers reported that they did not know what babywearing meant.

## Discussion

This study was the first to elicit the priorities for a psychological intervention aimed at reducing suicidal ideation and behaviour during the perinatal period, using a P-set consisting of mothers and perinatal mental health professionals. The centroid factor analysis identified two factors which provide insights into important elements to be considered when designing the content and delivery of a future psychological intervention. Support to help mothers distance themselves from the appeal of suicide through offering a greater choice of coping strategies was more important for mothers than professionals, whereas professionals and a small proportion of mothers prioritised helping mothers establish positive connections with the therapist, her baby and with motherhood more generally. Normalising the lack of a “rush of love” for the new baby, planning how to keep the mother safe and helping mothers cope with the urge to harm themselves were endorsed by participants that loaded onto both factors.

Factor 1 outlined a viewpoint that prioritised planning “for when the darkness descends/feeling absolutely nothing.” This statement was derived from the findings of a previous qualitative study in which mothers who had experienced suicidal ideation during the perinatal period reported feeling an intense darkness prior to a suicide attempt; the authors named this phenomenon “the darkness descends” (16). The extreme ranking of this statement suggests that participants recognised this phenomenon which has been described relatively minimally in previous research [e.g., (39)]. It also suggests that mothers fear that this feeling could (re)occur, so not only is planning for keeping the mother safe important as a way of responding to this feeling without engaging in suicidal behaviour, but an intervention should also aim to ensure this feeling of “darkness” does not return in the future.

Furthermore, there was consensus across the two factors that an intervention should help women to cope with the urge to harm themselves and that this intervention should include planning in response to suicidal thoughts when they are alone. Safety planning interventions, which may include ways to manage self-harm and suicidal urges through the creation of a structured and personalised resource which helps individuals identify an imminent crisis and use suicide-related coping strategies, are currently recommended for preventing recurrence of self-harm in adults by NICE in the UK (10). Two small pilot trials have shown safety planning interventions delivered to adults at risk of suicide, by a peer with lived experience of suicide (40) and a smartphone application (41) could result in fewer emergency department visits and reduced severity and intensity of suicidal ideation, respectively. These positive results are supported by the findings of a systematic review of 26 studies that examined the effectiveness of safety planning interventions based on that of Stanley and Brown (42) for adults who experienced suicide-related distress (43). The review authors found safety planning interventions were associated with improvements in suicidal ideation and behaviour, and reductions in participant depression and hopelessness. However, Nuij et al. (44) conducted a meta-analysis of six studies with a total of 3,536 participants that evaluated the effectiveness of safety planning type interventions in reducing suicidal ideation and behaviour. The authors found that safety planning type interventions were associated with reductions in suicidal behaviour but had no effect on suicidal ideation. Taken together, findings suggest that the inclusion of safety planning interventions are effective at reducing suicidal behaviour which aligns with the viewpoints identified in the current study. Therefore, extending and tailoring the safety planning intervention (42) to meet the needs of mothers during the perinatal period presents a promising direction for intervention development in the future.

Overall, Factor 1 endorsed helping mothers to psychologically distance themselves from the appeal of suicide, but why might mothers perceive suicide as an option in the first place? A mother’s ability to solve interpersonal problems may explain this, because it is theorised that diminished problem-solving limits the number of available adaptable solutions to a problem which increases the risk of suicide [e.g., (45, 46)]. Reduced problem-solving ability has been associated with suicidal ideation and behaviour in non-perinatal samples [e.g., (47–49)] and interventions that help individuals solve interpersonal problems have been recommended to reduce suicidal behaviour (50). There is a paucity of research that investigates poor problem-solving and suicide outcomes during the perinatal period, although Wagner et al. (51) examined correlates of suicidal ideation, including problem-solving orientation and problem-solving skills, in pregnant women living with HIV in Uganda. The authors found moderate or severe suicidal ideation was significantly correlated with greater use of negative problem-solving, a problem-solving orientation which is less effective in coping with psychological distress. Wagner et al. (51) recommended the use of evidence-based problem-solving therapy to manage stressors which could reduce suicidal ideation.

Factor 2 highlighted the importance of the therapeutic alliance when accessing an intervention and this was mostly prioritised by professionals. It begs the question of whether professionals overemphasised the importance of the therapist seeing as Factor 1, which was only loaded onto by mothers, did not see statements related to the therapist ranked as highly. We do not think this was the case because, firstly, three mothers loaded onto Factor 2 and therefore shared the view that it is important an intervention is delivered by the same therapist who demonstrates that they understand. Secondly, professionals were required to have worked in direct contact with and provided care to women who felt suicidal during the perinatal period for at least 3 months, whereas mothers were not required to have accessed any kind of mental health support to be eligible to participate. Hence, some mothers might have never received the support of a therapist and could not therefore draw on that experience to assist them in their judgement of how important the therapeutic alliance might be to the outcome of an intervention. Furthermore, a recent systematic review of 19 quantitative studies summarised the relationship between the therapeutic alliance in psychotherapy and suicidal experiences prior to, during and following psychotherapy (52). The authors failed to identify a significant association between suicidal experiences prior to, or during, the psychotherapy and the strength of the therapeutic alliance. However, the authors reported that establishing a strong therapeutic alliance early on in psychotherapy was related to reduced suicidal experiences in the future. An important recommendation in Huggett et al’s. (52) review was that therapists should dispel myths regarding the consequences of disclosing suicidal experiences and address any difficulties that clients might have when developing a therapeutic relationship. The application of this recommendation when working with clients during the perinatal period is particularly important because of mothers’ fears that their children would be removed from their care if they disclose suicidal ideation and/or behaviour to healthcare professionals (28, 53). As the Factor 2 viewpoint attributed great importance to the therapist, we also wish to echo the recommendation proposed by Huggett et al. (52) that future trials of psychotherapeutic interventions to reduce suicidal experiences should consistently measure both the client and the therapist’s perceptions of the therapeutic alliance.

Consensus statements demonstrated that both viewpoints endorsed normalising a mother not feeling a “rush of love” for the baby after birth which highlights that a mother’s perception of a relatively small event can have a huge impact on her mental health. A correlation between self-reported bonding impairment and suicidal ideation has been demonstrated (54) but as far as we are aware there is no literature that focuses on a mother’s immediate perceptions of bonding following birth and her mental health, which presents an avenue for future research. It is also important to consider that a heightened awareness of bonding difficulties could drive a mother’s fear that if she does not experience a “rush of love” as she expects, she might then interpret this as her having difficulty bonding with her baby. Therefore, an intervention that offers reassurances to mothers that her experiences are not in and of itself indicative of a problematic bond could help to undermine negative self-perceptions (i.e., being a bad mum) which could then, in turn, reduce the likelihood of suicidal thinking.

### Strengths and limitations

The inclusion of both mothers and professionals in this study provided a holistic insight into how the priorities of these two groups overlap, a major strength of this study. Furthermore, the two factors explained 42% of the overall variance, and Kline (55) advocated that a factor solution that accounts for 35–40% or more of the common variance is considered a sound solution. In addition, 25 of the 32 Q-sorts significantly loaded onto one of the factors, which suggests that the factor solution included a contribution from a large proportion of the Q-sorts. Another strength was the involvement of the study service user reference group, which helped develop the Q-set and pilot the Q-sorting task.

Although the two factors accounted for a considerable percentage of the overall variance, the factors were strongly correlated, which suggests a high degree of overlap between the viewpoints outlined by each factor. This overlap could be indicative of one of two possibilities: 1) mothers and professionals both held similar views with regards to what should be included in an intervention and therefore the findings provide a useful starting point for the development of an intervention in the future; or 2) sampling bias was present, and only mothers and professionals with a particular range of views on this topic participated. The use of several recruitment pathways aimed at ensuring a diverse sample of mothers and professionals to reduce sampling bias. Although, the sample of professionals varied in terms of the posts held, it should be highlighted that our sample of mothers lacked in ethnic diversity and were generally highly educated.

Only two of the mothers experienced suicidal thoughts at the time of participating in the study. We anticipated that a mother’s perceived priorities for an intervention might change as time passes after experiencing suicidal thoughts and after leaving the perinatal period and this is why we hoped to recruit a more equal number of currently and previously suicidal mothers; our failure to do this is a limitation of the study. In addition, three mothers did not know what the term ‘babywearing’ (see “encourage babywearing” (S68)) meant, therefore these participants’ rankings of this statement will have been affected.

### Clinical implications

Based on the viewpoints outlined by each factor array and the consensus statements, the design of a future intervention should focus on one-to-one therapy (rather than group sessions), prioritise planning to keep the mother safe and include problem-solving training for when a mother experiences suicidal thoughts alone and/or when “the darkness descends.” The content of the intervention should focus on providing a better understanding of how to identify and respond to thoughts that drive suicidal ideation and suicidal desire. The content should also challenge and explore how the mother perceives and feels about her baby and motherhood. The intervention should ensure mothers are seen by the same therapist who demonstrates understanding of, and attaches importance to, the fostering of a trusting therapeutic alliance. When considering how an intervention is delivered, attention and funding should be committed to ensuring childcare support is available for mothers trying to access the intervention.

The use of digital mental healthcare interventions is on the rise (56), despite research demonstrating a preference for in-person over digital psychotherapy for depression (57) and a high attrition rate of those accessing an online self-help therapy for suicidal ideation (58). Our findings emphasise the importance and irreplaceability of an intervention being delivered by a human and ideally in-person, for mothers experiencing suicidal ideation during the perinatal period.

### Implications for future research

For a psychological intervention to be efficacious, it must be theoretically derived and address the psychological mechanisms that underlie suicidal thoughts and behaviour (11). Future research should explore the psychological mechanisms pertinent to suicide during the perinatal period, which are hinted at by statements deemed important in this study. For example, the impact of a mother’s perceived self-efficacy on her suicidal thoughts and behaviour appears important because of the high rankings of “challenge the idea that the baby would be better off without their mother” (S22) and “challenge the thoughts of being a ‘bad mum’” (S23). Moreover, the agreement that normalising mothers not feeling a “rush of love” following birth suggests research should investigate mothers’ expectations of bonding with her infant after birth, her immediate perceptions of the bond after birth, and how these a) affect mother-infant bonding, b) affect her suicidal thoughts, and c) affect her perceived self-efficacy as a mother. Investigating problem-solving and its effect on suicidal ideation and behaviour also offers an avenue for future research.

Some mothers reported their hesitation at seeking support and because of this highlighted that the Q-set should have included a statement pertaining to addressing this problem. Although a separate issue to the content and delivery of a future intervention, this feedback demonstrates the urgent need to improve how mothers are encouraged to seek support and how mothers in need of support are identified; researching these improvements should be a priority.

At present, safety planning interventions are recommended as best practise by NICE (10) to prevent recurrence in those who self-harm. In this study, mothers and professionals converged on endorsing the importance of developing, and having available, plans to keep the mother safe and plans on how to support her through a crisis. Future research should investigate best practise and the effectiveness of safety planning to reduce future suicidal behaviour in mothers who experience suicidal ideation only during the perinatal period.

## Conclusion

In our study, both mothers and professionals believed developing plans to keep the mother safe from suicide when alone and in crisis was a priority for a potential psychological intervention to reduce suicidal ideation and behaviour. Mothers strongly endorsed the need for childcare support to be available while accessing the intervention, whereas ensuring a robust therapeutic alliance was generally more important for professionals. The findings provide an essential first step in the development of a new suicide prevention intervention for perinatal women.

## Data availability statement

The raw data supporting the conclusions of this article will be made available by the authors, without undue reservation.

## Ethics statement

The studies involving humans were approved by London – Brighton & Sussex Research Ethics Committee. The studies were conducted in accordance with the local legislation and institutional requirements. The participants provided their written informed consent to participate in this study.

## Author contributions

HR: Conceptualization, Data curation, Formal analysis, Investigation, Methodology, Project administration, Writing – original draft, Writing – review & editing. DP: Conceptualization, Funding acquisition, Supervision, Writing – review & editing. DE: Conceptualization, Funding acquisition, Supervision, Writing – review & editing. AW: Conceptualization, Funding acquisition, Supervision, Writing – review & editing.
